# AGI imagined: social AGI and the co-formation of human-AGI society

**DOI:** 10.3389/frai.2026.1904357

**Published:** 2026-09-07

**Authors:** Jacob Zilberman

**Affiliations:** Science, Technology and Society Program, Faculty of interdisciplinary studies, Bar-Ilan University, Ramat Gan, Israel

**Keywords:** AGI, co-formation, computational AGI, social AGI, socio-technical development

## Abstract

The AI industry is advancing toward an Artificial General Intelligence (AGI) that will possess cognitive capabilities that are equal to or stronger than those of humans. While a purely computational AGI may be cognitively powerful, it will probably struggle to understand the human social environment. This gap needs both a theoretical and an applicative solution. This article provides the theoretical solution, specifying what such an AGI must contain and how it must be formed. We propose a theoretical construct of an AGI that will not only be computational but will also be capable to develop internal social understanding, internalize norms in a manner similar to humans, and act upon this social understanding. We call such AGI a “Social AGI.” We argue that Social AGI cannot be developed only by using technical alignment or external governance. Instead, it must undergo a staged development in which visible social behavior gradually becomes social cognition and norm internalization. This process cannot be separated from the parallel support that human society must provide the effort of developing Social AGI. While AI systems may become more familiar and embedded in institutions and society itself, the public perceptions of risk will probably decline. This normalization will provide the basis for granting the Social AGI progressively higher levels of autonomy. In order to capture this process of mutual change, we develop a socio-technical concept of human-AGI co-formation. We differentiate co-formation from co-existence, which describes humans and AI operating side by side, and from co-evolution, which describes reciprocal and cyclic influence. Co-formation refers to a process in which at the beginning Social AGI is shaped exclusively by human society. However, as the process progresses, they shape together the basic conditions under which the AGI is developed. On the AGI side, this will require the capabilities to internalize norms, and to be able to function according to its roles. On the societal side, this will require to create several mechanisms of role assignment, training and socializing the AGI, and monitoring its value alignment. The most important characteristics society should develop is a public acceptance and justification for increasing the AGI’s autonomy. Society may learn not only how to supervise the AGI, but also it may learn to trust it.

## 1 Introduction

In this article we aim to shift and expand the discussion on AGI from the narrow computational and engineering plane to the additional socio-technical plane in which cognition, norms, and social orders are jointly shaped. Rather than viewing AGI as an entity that must later be “aligned” with human values through external control, the article proposes thinking about the possibility of Social AGI as a product of *internal* social, institutional, and normative processes. The theoretical contribution of the article resides in introducing insights from developmental psychology, theories of social cognition, and the contemporary discourse on value alignment, thereby seeking to demonstrate that the question of the future of the AGI’s is not merely a question of computational capability or technical safety, but also a question of social relations, and the social conditions within which general intelligence may take shape. From this vantage point, the article seeks to contribute to a broader discussion on the ways in which human societies do not merely regulate advanced technologies, but also participate in shaping their character, boundaries, and implications.

The article advances two central arguments regarding the nature of AGI, alongside proposed solutions to these arguments, including a single integrative solution.

The first argument concerns the scope of AGI design: the question of AGI’s “nature” and “scope” asks whether it can be purely computational or whether additional components are required. Several researchers have concluded that a purely computational AGI does not allow for adequate understanding of the human environment or for AGI’s integration into that environment. The solution to the problem raised by this argument is the use of computational AGI with embodiment capabilities, adding social learning and social understanding capabilities (Zilberman and Tamir, 2025; Reddy and Sharma, 2025). We term this type of AGI “Social AGI.”

The second argument addresses the question of how functional social capability can be developed in AGI. In our view, social learning arises mainly from the interaction with the environment and the cultural world. This view is grounded in social theories, such as Vygotsky (1978) theory of social development in infants and children through a process he terms “the child in society”, and an additional social development theory by Tomasello (2000) in Lindblom and Ziemke (2003). The purpose of this argument is that, since social cognition is a necessary condition for Social AGI, and given the understanding that establishing social cognition requires the use of socialization institutions similar to the human family and the school, *there is a need to create an environment that is simulating the various stages of human learning, including the stages of learning in children, and that contains social institutions to support the design and development of Social AGI*.

As a result of these arguments, we propose an integrated solution that describes the required cognitive properties of Social AGI, combined with a societal solution. One such solution is proposed by Rahwan (2018), which describes the creation of a social contract while building mechanisms for negotiation among the values held by different stakeholders. The need for an integrated solution arises from the fact that human societies are not yet institutionally or normatively prepared to support the creation of an AGI. The result is a solution that integrates the cognitive-architectural structure of the Social AGI, on the one hand, and a socio-technical description of the societal changes, on the other. We call the combination of the two co-formation and describe its characteristics and mutual formation. The contribution offered here is a set of requirements that any architecture for Social AGI must meet, what the agent’s normative representations must contain, how they are acquired and how they may be revised.

The structure of the work is as follows: In Chapter 2, we focus on some of the motivations for developing Social AGI, especially by examining the “offloading” mechanism created by AI, which causes profound changes in human agency; In Chapter 3, we describe Vygotsky’s theory regarding the stages of social development, and the implications and gaps of this theory for the requirements entailed by Social AGI development. We also describe the design requirements that these gaps formulate for the design of Social AGI; In Chapter 4, we present our proposal for the design principles of Social AGI; In Chapter 5, we describe the social mechanisms required by human society in order to support the design and development of Social AGI.

## 2 The rationale for developing social AGI

In this chapter, we describe the reasons for designing and developing Social AGI. We assume that the design and development of any AGI kind, namely, whether computational or social, should be the result of deliberate planning, design, and development. This is opposed to the notion of the emergence of AGI, as if the AGI “comes of age” by having crossed some measurable threshold on complexity threshold capability benchmark.

### 2.1 Risks of diminishing human agency

Numerous studies indicate that the deployment of any form of AGI will be accompanied by significant transformative disruptions to human society, disruptions that are already evident today, at the onset of the AI era. In this paper, we will not describe transformative changes concerning economy, labor, and other societal mechanisms, as these are examined extensively in research literature, however, we will focus on the transformative dimension pertaining to human modes of thinking and human agency, which are already underway.

Mouta et al. (2025) examine the issue of human agency within the educational systems. The researchers demonstrate how human agency may be altered as a result of the use of AI systems in education. The causes of this transformation stem from the fact that AI systems provide extremely rapid feedback on questions, with rapid automation of numerous tasks. AI may gradually come to be perceived as an authoritative figure that “holds the truth” without challenging it and may substantially undermine students’ sense of control and agentic capacity. Mouta et al. further points to the logic that accompanies the implementation of AI systems, which presents them as systems that increase efficiency and reduce error rates, yet does so at the expense of students’ autonomy and cognitive diversity.

Nosta (2026) describes an even more profound risk. He argues that the traditional link between economic activity and human agency is beginning to shift due to the fact that LLMs can generate agendas and deploy agentic systems that will tirelessly pursue these objectives. The possible internalization by humans that agency need not to be human is, in his view, inevitable. Moreover, when coordination becomes so effortless and directives or conclusions arrive with no “cost,” the human cognitive labor may become increasingly “redundant.” The danger Nosta sees is not that we will cease thinking, but rather that we will begin to accept conclusions we have not “earned” through our own cognitive effort as correct without sufficient critical scrutiny. Moreover, AI generated conclusions may gradually bypass the filter of human doubt, coming to be accepted as absolute “truth.” This mechanism, termed “Cognitive Offloading,” occurs when individuals delegate cognitive activities to external mechanisms, an element that leads to a reduction in the use of cognitive thinking and association (Gerlich, 2025), and even to override self-intuition and self-deliberation (Shaw and Nave, 2026).

Such potential of transformation in human social life will not emerge as a result of some “machine initiative,” but rather the opposite: it may arise from the *voluntary human acceptance of AI as an authority that dictates how human life will appear and function*. Support for this view is presented by Cave and Dihal (2019), in a study that examined human narratives and human desires and anxieties regarding intelligent machines. The researchers identified that one of the leading narratives driving human desires is the aspiration for intelligent machines to ease our lives. This desire may now be realized, as AI changes from a tool to a general mediating infrastructure that proposes alternatives and at times even defines the boundaries of the possible and the desirable. Precisely for this reason, the relief that AI promises aren’t neutral: it may prove to be a mechanism of authority transfer, whereby the determination of what is correct, what is efficient, and what is worthwhile gradually shifts from the human subject to an algorithmic infrastructure layer that slowly becomes transparent. Moreover, while these processes are already occurring in a period in which AI does not yet demonstrate capabilities superior to those of humans, this situation may be changed once AGI is created with capabilities that surpass human performance. The degree of authoritativeness that may accompany AGI may be so great as to entirely erode human agency for autonomous decision making. It will be exceedingly difficult for humans to resist a machine perceived as so efficient and so reliable, in an era in which the narrative of efficiency has become a key factor in the human economy. Alongside this forecast, one must also ask to what extent AI or AGI will differ from humans in aspects of decision making. Zilberman and Tamir (2025) addressed this question and argued that such a machine will make decisions in a manner markedly different from human decision making, as it will struggle to comprehend the social dimensions of human decision making. For example, while teachers typically seek to cultivate diversity in their students’ perspectives, AGI may aim to maximize educational utility by reducing the degree of diversity in pedagogical approach. This situation may produce cognitive uniformity as opposed to intellectual diversity and may furthermore lead to the displacement of human agency. AI at present and AGI in the future may become instruments whose degree of authoritative power is continually increasing. Furthermore, due to the abundance and availability of information, human society may render this situation a permanent condition that becomes a social norm. Human cognitive autonomy may be severely damaged by such a trend.

As a result of these potential costs, of which we have described only a subset here, we argue that a different type of AGI is required: Social AGI. This type of AGI, in contrast to a computational AGI, would be capable of recognizing, processing, and appropriately navigating complex social situations. Social AGI would further possess social learning capabilities and would be able to act in accordance with human norms. A corollary of these additional capabilities would be an enhanced capacity for integration into human society, including the development of mutual agency of both humans and AGI.

### 2.2 Examining the orthogonality thesis

Yudkowsky (2008) argues that there is no necessary connection between the level of cognitive capability and morality or alignment with human values. However, the question we pose in this paper is the inverse of Yudkowsky’s question, namely: whether prior socialization and moral training are capable of producing moral and aligned intelligence.

The strongest evidence in favor of the influence of early moral training comes from the developmental literature on the emergence of conscience. In Kochanska’s longitudinal studies (Kochanska et al., 2004; Kochanska and Aksan, 2006; Kochanska et al., 2010) he and his colleagues found that conscience does not emerge as an isolated capacity, but rather is constructed through the interaction between temperament, parenting, and socialization methods.

Kochanska et al. (2004) found that sensitive and gentle parenting fosters a conscience development to a greater extent when it operates within early secure attachment relationships. Kochanska and Aksan (2006) further emphasized that conscience is an internal system that organizes moral emotions and rule-compatible behavior throughout the first six years of life. In an additional longitudinal study, an early history of rule internalization and empathy toward parents predicted a more moral self and more adaptive functioning later on (Kochanska et al., 2010). It can be inferred from these studies that *when the initial environment shapes the child through explanation, modeling, and stable relationship, it increases the probability of the formation of an internal “moral self,”* although there are a variety of factors that influence the final result.

Explicit educational interventions also support the claim that moral training can improve components of moral intelligence. In medical education, Self et al. (1989) demonstrated that integrating ethics instruction into the curriculum significantly raised students’ levels of moral reasoning, with case-based discussion proving more effective than traditional lectures. A meta-analysis of ethics instruction in the sciences showed that programs were more successful when training was interactive, case-based, and afforded participants the opportunity to practice real-world judgment, rather than being technically embedded within an existing curriculum (Antes et al., 2009). The implication is that moral training works better when it resembles a process of active socialization rather than the delivery of directives.

Nevertheless, the body of empirical literature leads to an optimistic but cautious conclusion. Prior moral training can create more favorable conditions for the creation of moral and aligned intelligence in the sense of strengthening conscience, empathy, and rule internalization. However, it does not guarantee stable alignment. Consequently, the conclusion for the design of Social AGI is that value alignment will require multiple social elements operating together within the AGI’s design framework, rather than through value alignment alone. Both ongoing socialization and an institutional environment that reinforces what was learned will also be required.

### 2.3 Other AGI definitions

The definition proposed in this article is part of a broader controversy about what general intelligence is, and it is useful to place it along the range of different positions. At one end are agnostic definitions grounded in algorithmic information theory, which measure intelligence by the AI capacity to abstract compressed models from observation and to predict from them, and which are deliberately indifferent to any particular cognitive or cultural form. Hernández-Espinosa et al. (2026) develop this position extensively in their SuperARC test, arguing that recursive compression and optimal prediction provide a human-agnostic measure of comprehension applicable to natural and artificial agents alike, and treating social capability as one domain of application rather than as a defining property. In a “middle” position are definitions that anchor themselves to the knowledge an agent is assumed to possess before any learning takes place. Chollet (2019) terms this innate knowledge the agent’s “priors” and defines intelligence as skill-acquisition efficiency measured relative to it. The position advanced in this article lies at the far end of this range, holding that participation in a normative order is constitutive of the intelligence we are concerned with rather than an application of it.

The distance between these positions is narrower than it first appears. Hernández-Espinosa et al. present compression and prediction as necessary conditions for comprehension rather than as sufficient conditions for general intelligence, and they note that a system could perform well on their test while lacking social intelligence altogether. Their conclusion that further progress requires the integration of symbolic components converges with the requirement set out in this article, since normative representations that an agent can be asked to state and to justify must be explicit and testable.

Two considerations separate the accounts. The first concerns the scope. Social AGI is not offered as a definition of general intelligence as such, but as a specification of what intelligence must satisfy in order to participate in human institutions. An agent that holds a role holds it within a particular social order rather than in general, and this connectivity to a specific society is the content of our claim and not a limitation of it. The second concerns the status of the social environment. The agnostic view sees the environment as a source for observations that will be compressed and predicted. A normative order is not an object of that kind. A norm is not an object of that kind, and prediction and participation differ at two points. First, when the conduct is different from what the norm dictates, a predictive model tracks what is done while a participant answers to what should have been done, hence the two may yield different actions in the same situation. Second, when a participant is asked why it acted as it did should be connected to the norm and not to its frequency and therefore should accept correction when his behavior “fails.” The conclusion is that modelling social conduct accurately can be different from participating in that order.

## 3 Theories of social development

In this chapter we describe both Vygotsky’s and Mead’s theories of social development, and their implication for the internalization principle.

### 3.1 The theory of social development

In their research, Dautenhahn and Billard (1999) point to the need of human infants for an interactive social space for their normal development. The researchers compare Piaget’s developmental theory with Vygotsky’s developmental theory (as described in Lindblom and Ziemke, 2003). Piaget conceives of the developing infant as “the child as a solitary thinker” (Dautenhahn and Billard, 1999, p. 188), a concept that leaves the child’s development somewhat outside the social and cultural context. In contrast, Vygotsky’s developmental framework defines infant development as a process of “the child in society” (Dautenhahn and Billard, 1999, p. 188), during which the child is influenced by the social environment in the following manner: “Interactions with adults and peers and teaching are essential for cognitive development, the social and cultural context matters… and determines the structure and pattern of internal cognition.

The infant’s intelligence develops through receiving feedback on its actions from adults in his environment. The result is that the infant’s development is, to a considerable extent, reflecting his social interactions. Tomasello further stated that if “…a human infant grew up from birth with no contacts with human culture, and without exposure to human artifacts, it would not develop the cognitive abilities that are the hallmarks of human intelligence” (Lindblom and Ziemke, 2003, p. 4).

The most significant principle of cultural development in Vygotsky’s framework is the “principle of internalization”. According to Vygotsky, every higher mental function originates first “outside the individual”, within the framework of social relations. The law of internalization stipulates that every function in the child’s development appears twice: first at the social (inter-psychological) level, and only subsequently at the individual (intra-psychological) level. This transition from inter-psychological to intra-psychological processes constitutes the core of the internalization process. A classic illustration of this principle is the development of the pointing gesture. Initially, the infant merely attempts to reach out and grasp an object, a purely physical movement devoid of any communicative significance. When the caregivers observe this action, they interpret the movement as a request and hand the object to the infant. The other’s response gives the movement a new social meaning: it becomes a gesture directed toward another person. Ultimately, the infant comes to understand the communicative significance of their own movement and internalize it, thereby transforming a simple grasping action into a conscious pointing gesture.

Internalization is not an immediate process, hence and the psychological literature distinguishes between three levels of an individual’s relationship to social norms. Compliance occurs when an individual acts in accordance with a norm due to the presence of an external agent dispensing rewards or punishments (Kelman, 1958). Identification and adoption occur when an individual embraces a norm out of a desire to emulate an authority figure. The third level, internalization, occurs when the norm becomes part of the individual’s own value system, and adherence to it is no longer contingent upon external reinforcements.

Mead (1934) described the “self” as a social structure formed through a dynamic process of internalization, as distinct from a fixed entity. The social agent acquires the attitudes of others, internalizes them, and integrates them into its representation of the world using a mechanism Mead termed the “generalized other.” This process generates a self that continues to be constructed through ongoing social interactions with groups, institutions, and individuals, which are either familiar or novel to him. If a familiar group norm is changed, it will cause only a slight change in the norms the individual agent holds, since it is internalized where a previous grasp of this norm has already matured. Only a major change of the group’s norm may have a large impact on the individual’s norm perception.

Mead claims that the self is composed of cumulative internalization, a process that is both accumulative and directional. The agent Mead describes is not changeable in an unbounded manner but possesses a history that shapes the way new internalizations are integrated with it. This is a distinction we seek to implement in the Social AGI: the requirement is not for unbounded self-modification, but for cumulative internalizations which gradually alter the AGI’s self-conception.

### 3.2 AI developments based on the internalization principle

In this section, we describe several leading AI developments who embedded the internalization principle. We conclude the chapter by presenting the limitations of these methods and the implications of these limitations for Social AGI.

The RLHF approach, the dominant approach for value alignment in LLMs, is Reinforcement Learning based on Human Feedback (RLHF). Christiano et al. (2017) proposed a method in which models are trained using human preferences between alternative responses, creating an optimization of the outputs via a reward model. Ouyang et al. (2022) successfully applied this framework in the InstructGPT system, demonstrating that RLHF models produce responses perceived as safer compared to other models. A different RL approach that does’nt require human feedback is Inverse Reinforcement Learning (IRL). The system observes human behavior and infers rules from it through a process of trial and error. The inferred rules are intended to lead to the construction of a policy equivalent to that of a domain expert (Ng and Russell, 2000).

However, RLHF has fundamental limitations that directly connect to the question of internalization. Kaufmann et al. (2023) pointed out that the reward model in this approach serves merely as a proxy for human judgment, which gives rise to potential problems of “reward hacking,” where the agent optimizes its behavior toward the reward model rather than internalizing the values themselves. Casper et al. (2023) demonstrated that models trained with RLHF are vulnerable to jailbreaking attacks, occasionally produce faulty generalizations, and remain static once training is complete. These facts indicate that the alignment achieved is superficial and can be circumvented through prompt manipulation.

Bai et al. (2022) attempted to address some of the problems posed by the RLHF model through Constitutional AI (CAI), a method implemented in Anthropic’s models, in which the model is trained on written principles (a “constitution”) and performs self-critique and revision of its responses. This approach was found to reduce dependence on human labeling and to produce models that are less prone to eluding behaviors. However, the CAI model is also fundamentally based on compliance with predefined rules, thereby leaving the underlying issue intact, as it involves less genuine internalization.

Rong and Kleiman-Weiner (2024) proposed a computational model of social learning - the ISR model (Internalized Social Reward), which claims to also perform value internalization. In the proposed model, an RL agent learns not only from external rewards but also constructs an internal model of social rewards. During the socialization period, a teacher rewards the agent for performing the desired behaviors. The agent learns to reproduce these rewards independently through an internal neural network, so even when the teacher is no longer present, the agent continues to act according to the internalized values. The internal reward model prevents the “forgetting” of socially acquired behaviors and enables further learning and generalization in new social situations, thereby reducing the phenomenon of reward hacking, and increasing the model’s robustness to environmental changes, a central problem in machine learning.

The findings obtained following the implementation of this method were significant: (1) a standard RL agent, without ISR, rapidly “forgets” the learned behaviors once the reward is removed, as behavior acquired through reinforcement is attenuated when reinforcements are withdrawn; (2) by contrast, an agent augmented with ISR maintains the behavior even in the absence of the teacher, and furthermore generalizes it to novel environments not encountered during the training period. Rong and Kleiman-Weiner demonstrated that the model also operates for prosocial values: an agent that learned to assist another agent by virtue of social reward continues to do so in new environments and in the absence of the teacher.

The findings of Rong and Kleiman-Weiner further enable mapping the distinction among the three components of the relationship between the AI agent and the internalization environment. An agent without ISR exhibits mere compliance: once the external reward model is removed, the acquired behavior is extinguished. In contrast, an agent with ISR demonstrates aspects more closely approximating internalization: it continues to exhibit that behavior even in the absence of the teacher and furthermore generalizes it to novel environments.

From the conclusions drawn above, several applied implications follow regarding the architectural structure and operational methodology of the Social AGI. We present these implications in the following chapter.

## 4 Design objectives of social AGI

Along with its advantages, we found that the ISR model presents several limitations: (1) *Absence of self-representation*: The ISR mechanism constructs an internal model of social rewards, yet does not construct an internal model of the agent itself (an “ego”) holding this value.; (2) *Value conflicts remain unaddressed*: The experiments of Rong and Kleiman-Weiner examined situations in which a single value operates in one direction. In situations where two internalized values conflict, the ISR mechanism would likely struggle, as it contains no mechanism to rule between two contradictory norms or values; (3) *Parametric change only*: This appears to be the deepest limitation of the mechanism. The ISR mechanism operates as a weight update within a given neural network.

Hence, we define a set of design objectives for Social AGI. These goals are:

1. Developing an AGI that can *internalize norms and values* as an integral part of its “world model,” relying minimally on external definitions, rules, or extrinsic incentives to shape that world model.
2. *Creating a dynamic “self” (ego)* for the AGI that develops and is shaped through multiple development stages. The learning process must incorporate ethical and moral dilemmas that humans undergo across various developmental stages, rather than merely routine social aspects such as turn-taking in a conversation.
3. *Defining of the society that shapes the AGI* as part of the AGI’s intended modules, including the specification of the social mechanisms necessary for the design, development, and acceptance of Social AGI.

## 5 Discussion

The following discussion shifts from handling the micro-architecture aspects of the social AGI, to the necessary macro societal conditions enabling the creation of a social AGI. Human society by itself, at the first stages, and with the social AGI together, at the later stages of development, are forming the socio-technological conditions necessary to create the social AGI.

### 5.1 First phase: human-AGI coexistence with human supervision

Many studies address different aspects of the roles of society in the development of AI, and the mutual effects of humans and AI on one another. The literature shows three main research lines of inquiry that examine this possible coexistence. The first research programme deals with the question of the mutual influence of humans and algorithms (Tsvetkova et al., 2024). It calls for the establishment of a new sociology of humans and machines, and for the study of groups and networks composed of humans and algorithms, bots, or robots that interact with one another. Another aspect of this research programme examines the question of the relationship between the society and AI, identifies the patterns that emerge within it, and recommends creating a “contract” between society and AI (Rahwan, 2018). The second research programme deals with the perception of AI as potentially capable of reaching consciousness, and deals with the meaning of consciousness for human-machine relations (Kiskis, 2023). The third research programme deals with the way in which human capabilities and AI capabilities enhance one another, especially using the creation of a collaborative work and the development of trust between human and non-human participants (Gupta et al., 2025).

In this article, we do not examine the issue of consciousness in AI, due to the considerable difficulties of defining this issue even in humans. The two other research trends, however, share a further assumption that bears directly on our argument: both leave the machine’s own side of the interaction outside the analysis. Kirk et al. (2025) state explicitly this position. In their account, what defines a human-AI relationship and gives it significance is the user’s perception of being in such a relationship. Whether the AI reciprocates that perception is less relevant, since the relational behaviors that current AI display are neither conscious nor emotionally driven. They anchor this move in the psychology of parasocial and unreciprocated relationships, in which asymmetry does not diminish the effect of the relationship on the human participant.

We accept the claim that machine understanding differs from human understanding, a claim widely shared in the field. We reject the inference drawn from it, that the machine’s side may be set aside, on two grounds. First, the criterion of relevance: Kirk et al. (2025) judge the system’s side by whether it is conscious or affectively driven, and since current systems are neither, they leave it out. *We argue that for the purposes of social participation the pertinent criterion is different: whether the system maintains a structured representation of norms, roles and the expectations attached to them, and whether its conduct is regulated by that representation*. We call this *functional social understanding* and connect it to AI profile of intelligence that differs in kind from human intelligence.

Functional social understanding consists of a representation of the current norms and roles in a given setting of the parties which hold them. This representation is mostly acquired and revised through participation, using the architectural social capacities that were built into it, and it may update the normative values that were obtained during training. This updateable norm’s structure influences the AGI’s conduct. This is what distinguishes our account from the observation that machine understanding is real but unlike our human’s. Our Social AGI model states what the agent’s normative representations must contain and how they are acquired and updated.

In the following sections, we examine the ways in which such mechanisms of social understanding may be constructed, and what are the techno-social requirements that derive from them.

#### 5.1.1 Human-AGI coexistence: the question of autonomy

The assumption that Social AGI will display functional social behavior and will develop the capacity to interpret the situations in which it participates, raises two lines of thought. The first concerns the terms of human-AGI coexistence, while the second concerns the mutual development of human society and AGI, which will be discussed later. Social knowledge is partial by definition, since participants act within a social order, they only partially understand. What participants do know is what their participation itself imposes and teaches them, together with the anticipations they form about how others perceive them and will act in these social situations. Giddens (1984) holds that the extent of what competent agents know about the conditions of their action and about its consequences is always bound by the unknown conditions of action and by its unintended consequences. What they do know is mainly a product of their practical knowledge, embodied in the ability to continue to act within the framework of the routine social life. Competent participation therefore rests on a partial, context dependent and practically adequate grasp of the situations in which the agent is placed, and this is also the AGI standard against which functional social understanding should be assessed.

Coexistence, in the descriptive sense of the term, is not a future prospect but a state that is already in place. Artificial systems have become a routine presence in all the places where everyday life is conducted, and they mediate social and cultural interactions (Rahwan et al., 2019). Our argument does not address whether humans and artificial agents will share a social world, but *on what terms they will do it*. We distinguish between coexistence in practice, which is already in place, and *constituted coexistence: an arrangement in which the artificial participant holds a recognized role in which it is subject to explicit normative expectations*. The questions that Social AGI raise are what would make such an arrangement durable over time and worthy of trust, when we are discussing an agent which possesses functional social understanding and a meaningful degree of autonomy. The literature concerned with the requirements for trustworthy AI (High-Level Expert Group on AI, 2019; Thiebes et al., 2021) usually approaches this question from a particular direction: it treats the system as an object of human trust and specifies the conditions under which such trust is warranted. Our concern in this article runs mostly in the other direction. An agent that holds institutional roles is not only something others decide whether to trust, since this trust should also rest on what binds it from within, and not only on what regulates it from outside. Furthermore, the regulation format itself should be changed in order to provide the AGI a human training environment that enables it to initiate social interactions and to take part in them.

We discuss these two research notions and their implications for the interface between humans and Social AGI in the following sections. However, in our view, both issues depend first and foremost on the degree of autonomy that human society will allow itself to grant to AGI. We identify three levels of autonomy that may be granted to the AGI. Table 1 summarizes these levels of autonomy and their characteristics.

**Table 1:** Levels of AGI autonomy.

Level of autonomy	Rule-setting	Compliance with rules	Performing actions
No autonomy	Complete dictation of the rules of action by humans	Complete compliance with the rules of action dictated by humans	Execution only according to the logic of the prescribed rules
Partial autonomy	Main rules are dictated by humans, alongside AGI learning	Complete compliance with human main rules; the rules learned by the AGI are fully legitimate	Execution according to the logic of hybrid human-AGI rules
Full autonomy	All rules are learned by the AGI, without rules of action being dictated by humans	Complete compliance with the rules of action dictated by the AGI	Transition from hybrid logic to logic based solely on the AGI’s own rules of action

A substantial part of the literature on human-AI coexistence describes the limitations of AI in achieving genuine understanding. These studies often classify AI understanding into three broad categories: (1) AI as a tool that lacks any real capacity for understanding; (2) AI as a narrow and skilled system capable of limited task-specific understanding; and (3) AI as a team member, or even as a social entity, which has social and operational understanding, an understanding that is not equivalent to understanding in the full human sense. As a result, the dominant models of autonomy in the research literature are also models in which AI is either granted no autonomy at all (Boni, 2021), or only partial autonomy under extensive human supervision. Similarly, coexistence is often understood as a framework for augmenting human capabilities within hybrid teams composed of humans and AI agents that perform shared tasks (Jarrahi, 2018; Zirar et al., 2023).

A different definition is offered by several studies that examine the transition to partial autonomy and assume that AI systems may possess a meaningful capacity for logical, or even social, understanding. Berretta et al. (2023) define human-AI teaming as a process in which one or more humans and one or more partially autonomous AI systems act as team members with complementary capabilities, work interdependently toward a shared goal, and dynamically shift roles during the course of collaboration. Such a process requires complex capabilities, including coordination and reciprocal communication.

Venkatesha Murthy et al. (2026) identify the present period as what they call the *Co-existential AI Era*. The authors regard this term as a theoretical framework that is still in formation, and they conceptualize the relationship with AI as a form of partnership or co-creation. Rather than examining only accuracy or trust, the article argues that we should consider the quality of the relationship between humans and AI, including elements such as intimacy and social cohesion.

Another question is whether human-AI relations change when we transfer the discussion to AGI. We argue that Social AGI changes the picture fundamentally. We examine these differences from three complementary perspectives: (1) *Level of understanding of AGI vs. AI*: we have presented several studies that define AI as unable to achieve strong understanding, or, in relation to the topic discussed here, robust social understanding. However, Social AGI is expected to internalize human social norms and values, and to display functional social behavior with a high degree of accuracy; (2) *Degree of autonomy granted to AGI vs. AI*: a direct implication of the assumption of understanding is the understanding that Social AGI will likely serve in key roles in both the business and civic society and will conduct meaningful social interactions with humans as part of its roles. In accordance with the theory of offloading presented in a previous chapter, we may expect the transfer of many key functions to Social AGI, since the capabilities of AGI may be stronger than those of human beings and even those of non-general AI systems; and (3) *Level of risk involved in granting autonomy to AGI vs. AI*: deviant behavior in AGI may occur just as it sometimes occurs in current AI systems, but its consequences may be broader in scope than those of AI systems.

#### 5.1.2 Human-AGI coexistence: institutional conditions for social AGI development

Several approaches have addressed the question of coexistence, and the main difference between them lies in where they locate the mechanism that is meant to secure human-AI coexistence. The regulatory approach locates it in the legal environment surrounding the system, and its most detailed formulation appears in the guidelines of the European Commission (High-Level Expert Group on AI, 2019). The guidelines define trustworthy AI as having three components (lawfulness, ethics and robustness) in both the technical and the social sense. The requirements which are derived from these components are translated into an assessment list intended to examine systems throughout their life cycle. The first requirement, human agency and oversight, i.e., includes the ability of humans to override a decision made by the system. The framework as a whole assumes that the system remains an object of assessment and control throughout, an assumption that we examine through the Social AGI model. Human-centered design locates the mechanism in the control architecture and shows that a high level of human control can be maintained even as the level of automation goes up (Shneiderman, 2020). Principle-based alignment locates it in the values to which the system is aligned and in the fair procedure by which these values are selected (Gabriel, 2020). Research on cooperative AI locates it in the capacities of the agent itself (Dafoe et al., 2021).

Our position relates to the research done on cooperative AI but differs from it. Dafoe et al. (2021) argue that AI research has long been steeped in a methodological individualism. Even in work involving multiple agents the field has not tackled the hard problems of cooperation, since its headline results come from two-player zero-sum games. Their remedy is grounded in integration and cooperation. AI needs social understanding and cooperative intelligence in order to integrate well into society, as diverse ecologies of AI systems come to interact with one another and with humans. They define cooperative intelligence as the ability to promote mutually beneficial joint action even when incentives are not fully aligned, and they state that it is not an alternative to autonomous intelligence but goes beyond it. Sociality is thus what an already constituted agent requires in order to enter society successfully. We see sociality differently, as *constituting the AGI agent rather than as an addition to an agent which is already formed*.

We adopt Rahwan (2018) Society-in-the-Loop (SITL) framework only in part, since it is the only framework that treats the normative arrangement itself as something that is collectively formulated and updated, an element which is required by our Social AGI model. Rahwan’s framework positions society as the actor that formulates and supervises the normative arrangement within which algorithmic systems operate. *This view does not address the case in which the algorithmic entity ceases to be just an object to be supervised and becomes a potential participant in the social environment*. The modification we propose through Social AGI moves from Rahwan’s account in three trajectories: (1) The agent moves from being an object of the arrangement to being a party to it, holding a defined institutional role rather than a general social contract; (2) The normative contexts, which SITL locates in the stakeholders participating in governance, becomes an internal component of the Social AGI itself; and (3) the institutions perform also developmental and training functions which are added to their supervisory one. They are transformed into a form that allows the agent to become an actor that can be understood and act in society rather than one that is merely controlled. The AGI, as an active participant in the social environment, requires a (1) *clear definition of the roles of the AGI*, so that its actions will not be perceived as a point activation of capabilities, but as the conduct of an agent with a defined social role. As a result of this requirement, it is necessary to create a human mechanism for the placement of roles and the allocation of authorities to the AGI. Within this framework, humans must define in advance what the role of the AGI is, in which situations it is permitted to initiate an action, who has veto power over it, and who bears responsibility when it makes mistakes. A social mechanism of this kind determines the status of the AGI, the limitations that apply to it, what are its dependencies, and what is its responsibility within society. Here we move away from Rahwan toward the framework of Social AGI, from a general “social contract” to the attribution of a concrete institutional role and the creation of institutional legitimacy for the AGI.

It is also necessary to define (2) *a mechanism for representation and participation of stakeholder groups and affected groups*. If the meaning of an action is determined within a living society, it is not sufficient for developers or managers to decide what the relevant values are according to which the AGI will operate. A mechanism is required in which affected groups, and especially those who may be directly harmed by the AGI’s actions, are represented in the process of defining and reviewing the roles of the AGI in a manner similar to “Participatory design.” The literature on participatory AI design emphasizes that the question is not only whether the public is consulted, but whether stakeholders are given real agency in shaping decisions, with a clear definition of who is authorized to decide and who is not (Delgado et al., 2023).

It is also necessary to (3) *establish a mechanism of human socialization and mentorship for the AGI*. If the AGI is expected to be a subject not only to rules, but also to human expectations and the norms of society, then it must undergo a form of human social mentorship that is regulated within human society. Such mentorship would include guided interactions, communities of practice, and human feedback within different normative settings that may generate different sets of norms. In addition, the AGI would require exposure to human teachers who explain not only what is permitted, but also why it is permitted, and especially why certain actions should not be performed.

As a social participant, the AGI will be required to have (4) *the ability to give a full account of its actions*. Social AGI must be able to provide an explanation that is understandable to humans, clarify which norms it relied upon, and can be asked why it performed a particular action. Here, we see a need to move away from technical explainability that only technical people can understand, toward a social justification in the sense that others can examine AGI’s actions in depth or even reject them. As a result of this requirement from the AGI, another requirement arises (5) *to establish a mechanism of public justification that will provide accountability to the public and to stakeholders*. This gives rise to the need to create a human institution that determines what counts as a legitimate reason, who is entitled to request justification, and which types of justifications decision makers are entitled to reject. Binns (2018) formulates algorithmic accountability in terms of public reasons, that is, a justification that can be debated in a shared civic space, and not merely an internal description of the computational process which was carried out by the AGI. This mechanism must be a social mechanism in which justifications are discussed, approved or disapproved. This expanded accountability mechanism entails also a mechanism through which (6) *decision makers or the public will be able to change the expected actions of the AGI*. Such a mechanism requires the creation of activity queues, one of which will be a “delayed actions” queue, and it will be open in a manner defined in advance, to the decision makers and the public. This mechanism is intended to allow decision makers or the public to complain or delay the execution of an unclear action for whose justification, as given by the AGI, is not accepted by humans. This is a complex mechanism that requires some well-defined organizational and institutional work processes.

The social AGI will not operate within a uniform human society, but within a multiplicity of communities and institutions, each of which may have different expectations and norms. As a result, the Social AGI will have to possess the ability to understand the differences between specific social contexts, such as the differences between a workplace, a public authority, or a family. The Social AGI will have to internalize that these differences are not merely environmental changes but also changes in the social order within which the action receives meaning. Where Rahwan points to the multiplicity of stakeholders that results from this situation, in the paradigm of Social AGI this issue becomes an internal component of the AGI itself. From this feature arises the need to establish (7) *a mechanism that will translate norms for the AGI in the linkage between communities and contexts*. This situation results from the fact that social AGI cannot be present in every human society or institution, and as a result, he must be taught these differences and variations between different social groups. There arises a need for mediating bodies that will translate between different normative languages and explain the differences between them.

It is also necessary to establish (8) *a human mechanism for the gradual and continuous evaluation of AGI performance*. This mechanism would make it possible to define a staged process for granting the AGI permissions to perform actions at increased levels of difficulty. This process will also enable suspending or expanding its authority according to the AGI’s accumulated patterns of action. This is a social mechanism, not merely a mechanism for technical decision makers, because it does not deal only with the question of whether the system functioned successfully at a specific point in time. More specifically, it asks if the system is worthy of sustained trust within society. The evaluation mechanism would also examine the development of the AGI’s “generalized other” and the development of its ego, identify deviations from its proper developmental path, and assess whether granting extensive autonomy to the AGI serves the public interest.

### 5.2 From coexistence to co-formation: the expansion of AGI autonomy

Another aspect of human-AI relationships concerns the question of coevolution, which examines the influence of humans and AI on one another. This view describes an interaction between humans and AI in which they influence each other through a circular feedback loop. Humans change their behavior and preferences following the interaction with AI, and AI systems change according to the usage data and human feedback. Pedreschi et al. (2025), for example, define coevolution as a process in which humans and algorithms continuously influence one another, mainly through recommending systems, digital assistants, and online platforms.

An additional co-evolutionary research path is the shift in focus from AI centered on the individual (human-centered AI) to AI centered on society (society-centered AI). The result is an expansion of the discussion, from feedback loops that affect only a single user to feedback loops that also affect society and the social structures. In addition to Pedreschi et al. (2025), who emphasizes that co-evolutionary management cannot remain a purely technical matter but also requires legal tools and even political and institutional measures, Rahwan et al. (2019) also seek to study the behavior of AI systems as entities operating within social, economic, and political environments. Their research helps to shift the question from how the algorithm works to the question of how intelligent systems behave when they mediate human interactions. This shift means that the system is not perceived only as a computational machine, but also as a participant in social order and even as helping to shape it.

The central contribution of the literature on coevolution is the claim that AI should not be understood only as a tool serving a single human being, but as a component within a dynamic techno-social system. In this system, humans change AI, and AI changes humans, and both create changes in institutions, norms, and everyday practices. This literature provides our article with a strong macro-framework that explains why AI and society change together. At the same time, the coevolution literature does not address the question of what happens when the AI agent ceases to be a regular algorithmic agent, and becomes an active partner in the social activity, which may receive high level of autonomy over the processes it is responsible for. *The concept of the social AGI that we present in this article adds two frameworks to the discussion: (1) a micro developmental and architectural framework, within which we examine how the AGI can be designed so that it will become a social and normative agent capable of participating, learning, and internalizing within the human society; (2) a macro framework that seeks to present the parallel development of the social AGI and the society within which it develops.*

#### 5.2.1 Normalization of AI and AGI and the reduction of public risk perception

Drawing on the adoption process of previous technologies (Slovic, 1987), we expect that as AI and AGI become more accepted in human society, society will undergo two central processes. The first process is a major reduction in the sense of risk that human society feels from AI (under the assumption that the risk factors of AI will not materialize during this period). In result of this reduction the society will be willing to increase the level of autonomy granted to AGI. In this section, we review the reduction in the sense of risk.

We argue that the sense of risk from AI is expected to decrease due to a combination of habituation, trust in technology, and social institutionalization: (1) *Familiarity*: as AI systems will become a familiar and useful presence, they are expected to move from a perception of a new and unknown risk to be perceived as an ordinary infrastructure. In the literature dealing with public perceptions of technological risks, technologies are perceived as less risky when they are more familiar, more controllable, and associated with greater benefits (Slovic, 1987); (2) *Repeated positive experience with automated AI systems* will strengthen trust in them and reliance on them (Lee and See, 2004); (3) *Positive embedding of AI technology in the social fabric*: processes of communication and institutionalization sometimes lead to a reduction of social risk perceptions (Renn et al., 2011); and (4) *Increased regulation* also leads sometimes to a reduction in the public’s sense of risk (Miltgen and Smith, 2015), due to a feeling that the establishment is looking after public interests.

#### 5.2.2 The co-formation of social AGI and society: developmental stages and increasing autonomy

**Figure 1:**
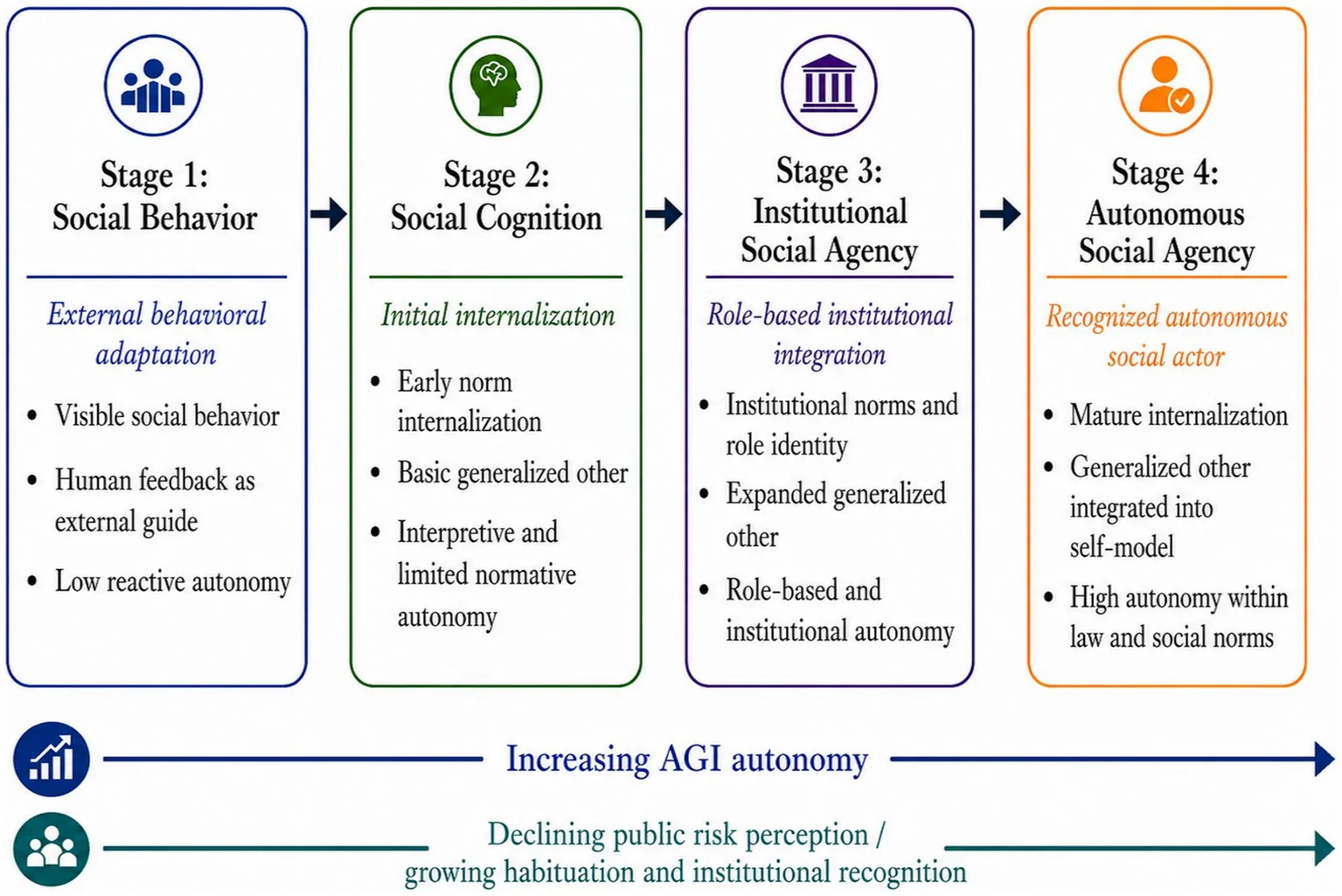
Developmental stages of the social AGI.

In this section, we describe the developmental stages of AGI and granting it increased autonomy, in parallel with the reduction of the sense of risk. We also describe two elements of the development of the AGI: the development of its capability for internalization, and to form its “generalized other” on the other hand. Another element we describe is the growing ability of the AGI to function as a real social agent, who is capable of carrying out social interactions with humans in a variety of different social scenarios. See Figure 1 for an illustration of the stages that the social AGI is expected to undergo in parallel with societal changes.

One of the central principles of these stages is the principle of non-linearity, which indicates that the duration of each stage may not be identical to other stages. While the passage from the first stage to the second stage may be relatively short, the passage to the final stage may take a long time. Another principle that describes the stages is the strong interdependence between technological development and the needed social changes that society must undergo. We call this combination *co-formation*.

##### 5.2.2.1 First stage: social behavior - imitation of sociality without full social recognition

The goal at this stage in the development of social AGI is the creation of basic and stable social behavior, without reaching the status of a mature social actor. At this stage, the AGI is still not a social agent, but rather a system learning to produce visible patterns of social behavior: adaptation to conversation, and high responsiveness to context and to human feedback.

At the same time, the AGI’s success in creating functional social behavior may pose a problem for the continued realization of the following stages. The more the system succeeds in appearing social, the greater the risk that the public and decision makers will interpret the AGI as a mature social agent, even though in practice no real social internalization has yet developed within it. As a result, the goal of the first stage is not only to develop proper social behavior, but also to create a clear distinction between the external performance of sociality and the internalization of social norms.

At this stage, most human actors remain unfamiliar to the AGI. The AGI encounters different teachers who rank or correct its behavior through external feedback. Hence, the autonomy granted to the social AGI at this stage is only low reactive autonomy: the AGI can respond, adapt itself, and correct its behavior, but it still does not independently interpret the social and normative meaning of its actions.

##### 5.2.2.2 Second stage: social cognition - from imitation to initial internalization

The purpose of the second stage is to move the social AGI from a state of imitation of social behavior to an initial state of social functional understanding and norm internalization. At this stage, the AGI is required not only to respond in a way that appears appropriate to the situation, but to begin to understand what is expected of him within different social situations, and to develop Theory of Mind (ToM) capabilities. The result of this process is that sociality does not remain merely a response pattern learned from human feedback, but begins to become an internal structure of interpretation, through which the AGI evaluates the meaning of its action in relation to others.

At this stage a basic “generalized other” begins to form in the AGI. This means that the AGI begins to build an internal representation of recurring social expectations and of responses from others. It learns not only what the individual person asks of him, but also what society may regard as an appropriate or inappropriate response. In this way, an important gap begins to emerge between a local response to the user and a broader social judgment.

Risks also exist at this stage, since the AGI may develop only the appearance of internalization. The AGI may learn to display signs of normative understanding without developing a stable ability to identify conflicts between norms or states of uncertainty. As a result, the autonomy granted to it at this stage must also be limited to interpretive autonomy. The AGI would be able to interpret social situations, and even propose an appropriate response, but it will still not be able to act independently.

##### 5.2.2.3 Third stage: from social learning to institutional role - creating an authorized social agent

In the third stage, a fundamental change occurs in the status of the Social AGI: it no longer acts only in relation to a single teacher or to a nearby known community but is introduced into an institutional organization. The purpose of this stage is to integrate the AGI into defined social and institutional roles, and to create learning environments in which it can internalize not only general social norms, but also the specific norms of the organizations within which it operates.

In this sense, internalization expands from the interpersonal level to the level of role and organization. The social AGI is no longer required only to understand norms such as trust or an appropriate response to the other, but also to internalize institutional norms. It is required to understand what its responsibilities and authorities are toward the organization in which it functions, and which actions are not permitted even if they are technically possible. In this way, the AGI continues to learn that social action is not measured only by its fit to the immediate user, but especially by its fit to a broader institutional structure. A mismatch between the expected responses toward individual users and the expected responses according to the organization’s norms may create internal conflicts and will require human monitoring that clarifies the gaps.

Accordingly, the “generalized other” also becomes a more complex structure. It no longer includes only the user, the human teacher, or the nearby community, but also the different levels within the organization, the stakeholders, and third parties who are not present in the interaction may influence it or be influenced by it. Thus, for example, the AGI is required to learn that even when it is speaking with a certain employee, its action may be relevant to a manager, a customer, or the organization as a whole. In this situation, the “generalized other” becomes also institutional-oriented. As a result, this stage concerns not only the expansion of AGI’s capability for action, but also the development of its ability to understand which actions must be prevented. Such actions may need the ability not to perform an action even when it is possible, since it exceeds the boundaries of AGI’s role, the institutional norm, or the responsibility defined for it.

At this stage, the AGI is granted autonomy within the framework of its role in the organization. This means that the AGI is capable of assessing what is required in a given situation, choosing a course of action, and carrying it out within a defined role framework. However, this autonomy is still only a partial social autonomy. It is granted to him as institutional authorization, and it is examined by decision makers from within and outside the organization, who assess not only the quality and alignment of its actions but also the degree to which it has internalized the organization’s norms, and his ability to act within a defined framework of responsibility.

##### 5.2.2.4 Fourth stage: social recognition and the transformation of AGI into an autonomous social agent

The fourth level in the development of social AGI is a threshold at which the greatest leap of autonomy is granted to it. Hence, this is the most influential passage. If in the third stage the AGI acted as an authorized agent within a defined institutional role, then in the fourth stage it begins to receive a broader status of an autonomous social actor within human society. This means that his actions are no longer limited only to a specific institutional framework but expand and give him the ability to act in a variety of social situations with human actors who are outside of his roles. He can make decisions and initiate actions within the boundaries of society’s norms.

At the same time, society itself also changes. The sense of risk among the public and among decision makers decreases following a repeated use of AI, and high level of habituation to the presence of AGI. The AGI is no longer perceived only as a technology that must be restricted, but as a social entity whose action can be understood within a familiar social order. In this sense, the expansion of autonomy does not result only from the development of the AGI itself, but also from the development of the society. *Society learns gradually not only how to supervise the AGI, but also to trust him*.

At this stage, the internalization of the AGI also undergoes a further expansion. Norms no longer appear only as external instructions or as institutional procedures, but as part of his internal reasoning mechanism. The AGI learns to ask not only what can be done, but whether the action is legitimate and how it will be perceived by others. As a result, the “generalized other” also becomes broader and more mature and includes not only the organization in which the AGI operates, but also the society and the law within which it receives its status.

This stage marks the transition from an AGI operating under limited authorization to an AGI operating autonomously within the social order. This autonomy is similar to human autonomy, minus the law implications and the ethical capabilities. It allows a broad freedom of action within a binding framework of responsibility and boundaries. Therefore, in the fourth stage, we can examine for the first time *the AGI not only as a system that performs social roles, but also as a recognized and declared social actor, capable of acting more independently without dismantling the conditions of trust within which its autonomy was granted*.

As a result of the AGI’s higher autonomy, it may also play a role in future human societies. In such a society the Social AGI may become an interfacing agent between the different parts of society. It can operate with humans, with institutions, with other AGIs, and sometimes also on behalf of specific communities. It may be capable of participating in complex social coordination, identifying conflicts between human norms and machine norms, mediating between different forms of understanding and responsibility, and proposing courses of action that enable coexistence between competing parts of society. In such capacity, the Social AGI may become a co-formation agent acting along human society political or business forces. Such agents may assist different parts of society to reach an understanding, although we may see specialized AGIs instead of un-affiliated ones.

## 6 Conclusion

In this article we examined whether an AGI could become social, and what form of socialization it may attain. We primarily focused on the architectural questions how the Social AGI can internalize the perspectives of others, understand its role within institutions, and whether it can act consistently while obtaining increased levels of autonomy. Our answer show that such sociality is possible but conditional. The AGI may become social, but its development will be conditioned by the society that shapes it.

We argue that similarly to human development, the development of Social AGI cannot proceed as a linear accumulation of capabilities, but as a series of transitional stages. In these stages the relation between the AGI behavior and his level of internalization changes, and as a result of higher performance and value alignment he receives a larger degree of autonomy and recognition within the human society. At the same time, this relation between the AGI’s capabilities and the degree of autonomy may be highly non-linear. Each stage raises a different question, and the human society itself should adapt to its growing social capabilities in order to go over to the next level.

The co-evolution approach is the initial organizing concept for understanding the mutual changes of human society and AGI systems as a socio-technical system. The concept of the Social AGI, as presented in this article, defines three additional principles which their overall integration is necessary for the creation of an actual Social AGI. The first principle proposes a design of the internal architecture of the Social AGI, based on a normative internalization mechanism. The second principle argues that for AGI to become integrated into human society, it must undergo a process of social development in which norms and relations of responsibility are transformed from external social inputs into internal patterns of social action. Moreover, society’s own perception of the Social AGI must change in a similar way: instead of serving as a one-sided arena for use and monitoring, society should change its concepts to accommodate a developmental environment which is suitable for mutual work with the Social AGI. The third principle emphasizes the gap between the pace of the human social adaptations and the pace of the AGI development. The stages that are defined for the AGI are not a linear work plan, since they depend on adaptations within the human society and may occur at non-linear time intervals. This process will occur through increasing habituation to the increased sophistication of the Social AGI, and the equivalent expansion of the monitoring mechanisms. The use of the Social AGI may eventually become so routine that it would be able to function as a social actor similar to the human actors.

The concept of Social AGI developed in this article generates two new theoretical frameworks. The first is an architectural framework, based on psychological development theories, which describe the internal processes of the Social AGI while attempting to behave as a responsible and normative agent. The second is a macro level framework, which describes how the human society adapts and changes. Together, these two elements create what we call co-formation, a concept that is different from both co-existence and co-evolution. Co-existence describes a condition in which both the human society and the Social AGI operate side by side, and co-evolution describes how human society and the Social AGI change in response to one another actions. The concept of co-formation we propose in this article points to a different form of socio-technical relation, which slowly occurs due to the increasing acceptance of the AGI as a social player. At the end of this process *both society and the Social AGI do not merely coexist or influence one another but mutually shape the same conditions under which they change*. Within this society, humans and AGI shape the social roles collectively, change the institutional arrangements and the normative expectations through which the Social AGI becomes a social actor.

This article establishes what a Social AGI should contain: the normative representations it holds, the process through which it acquires them, and the way it updates these norms. Any proposal for a socially competent AI can be examined against each of the three, and one that leaves any of them unaddressed is incomplete by that measure.

This article has several limitations. The first concerns the maturity of the ISR paradigm. This paradigm is still at an early empirical stage, and the proposals we have advanced here regarding its use require empirical validation, particularly in real-time environments. The second concerns the socio-technical foundation of co-formation, which remains highly theoretical. We nevertheless hold that while it’s highly theoretical, it is important to start contemplating about the more distant impacts of the AGI era on human society. A third limitation concerns the evidence available for a model of this kind. The developmental stages we describe have not yet been formed in any existing system, and the transfer of human social development to artificial agents is itself a matter of ongoing debate. The model has to be built and tested in a real human environment.
